# 149. Determining the Feasibility of a Rapid Test in Detecting the Presence of the Cefazolin Inoculum Effect (CzIE) in Methicillin Susceptible Staphylococcus aureus (MSSA)

**DOI:** 10.1093/ofid/ofaf695.051

**Published:** 2026-01-11

**Authors:** Jacob Dziadula, Diana Panesso-botero, Kavindra Singh, Paola L Carvajal, Sandra Rincon, Jinnethe Reyes, Asmita Ghosh, Marissa Schettino Intriago, Erika Flores, Sharon Lechtig-wasserman, Wesley Long, Patricia L Cernoch, Benjamin Howden, Steven Tong, Hon FRACP, Jose M Munita, William R Miller, Cecilia Tran, Cesar A Arias

**Affiliations:** Houston Methodist Research Institute, Houston, Texas; Houston Methodist Hospital, Houston, TX; Houston methodist research institute, Houston, Texas; Universidad el Bosque, Bogota, Distrito Capital de Bogota, Colombia; Universidad El Bosque, Bogota, Distrito Capital de Bogota, Colombia; Universidad El Bosque, Bogota, Distrito Capital de Bogota, Colombia; Houston Methodist Hospital, Houston, TX; Houston Methodist Research Institute, Houston, Texas; Houston Methodist Research Institute, Houston, Texas; Houston Methodist Research Institute, Houston, Texas; Houston Methodist, Houston, Texas; Houst, Houston, Texas; Microbiological Diagnostic Unit, Peter Doherty Institute, Melbourne, Victoria, Australia; Doherty Institute, University of Melbourne; Instituto de Ciencias e Innovación en Medicina, Santiago, Region Metropolitana, Chile; Houston Methodist Research Institute, Houston, Texas; Houston Methodist Hospital, Houston, TX; Houston Methodist and Weill Cornell Medical College, Houston, TX

## Abstract

**Background:**

Infections with *S. aureus* continue to be a significant clinical burden. While cefazolin (CFZ), a first-generation cephalosporin, remains a key therapy against MSSA bloodstream infections (BSIs), some studies have suggested poor outcomes when CFZ is used in the presence of the cefazolin inoculum effect (CzIE). However, broth microdilution at standard and high inoculum, the gold standard test for detection of CzIE, is impractical for clinical microbiology labs. In this study, we evaluated the feasibility of rapid colorimetric testing using nitrocefin (NCF) to identify the CzIE in real-time in a clinical microbiology lab.

**Figure:**
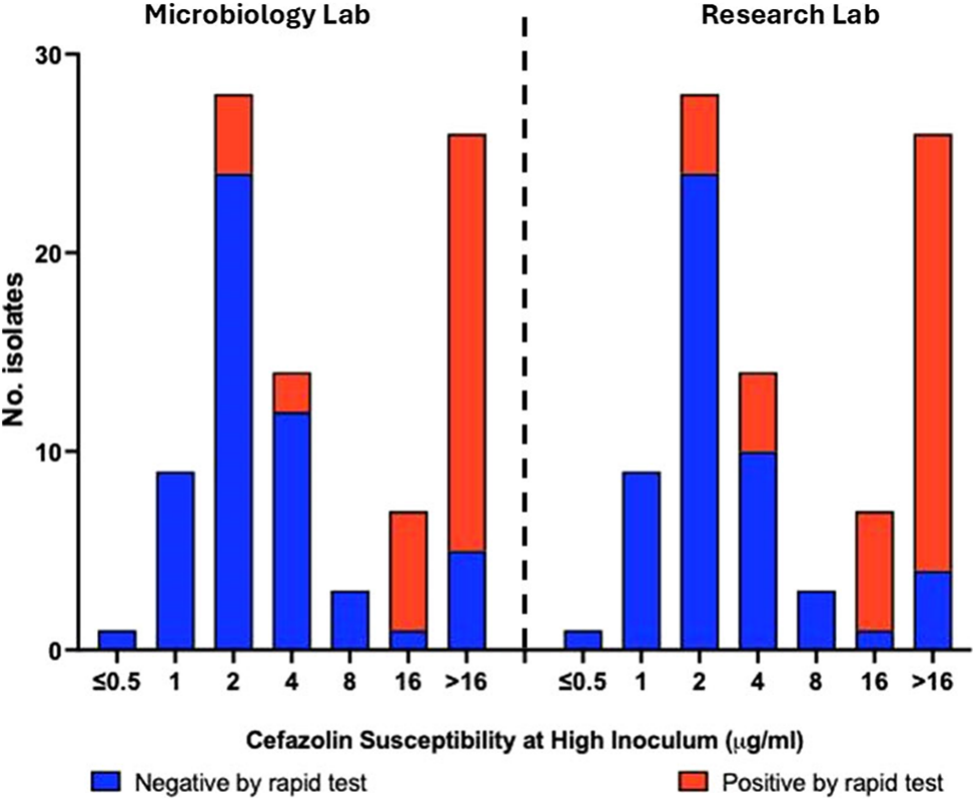
CzIE by MICs Rapid test results stratified by MICs when comparing real time performance in the micro lab versus the controlled environment of a research lab.

**Methods:**

Eighty-eight MSSA isolates were recovered from initial cultures of adult patients between January 2025 and April 2025 at the central lab of an urban hospital system. The NCF-based rapid test was performed in the clinical microbiology lab in real-time according to a published protocol. In the research lab, the CzIE was then assessed using broth microdilution (BMD) at standard (10^5^) and high (10^7^) inocula, with a cutoff CFZ MIC ≥ 16 μg/ml. The NCF-based test was repeated at the research lab under controlled conditions. Sensitivity, specificity, accuracy, and concordance rates were calculated.

**Results:**

Thirty-three (37.5%) of the 88 isolates were positive for the CzIE by the BMD method. Using the NCF rapid test, 33/88 (37.5%) isolates were determined to be positive for the CzIE in the microbiology lab, compared to 36/88 (40.9%) in the research lab. The overall accuracy of the real time rapid test performed in the micro lab was 86.4% (76/88) with a specificity of 89.1% (49/55) and a sensitivity of 81.8% (27/33). When the rapid test was performed in a controlled environment, it had an overall accuracy of 85.2% (75/88) with a specificity of 85.5% (47/55) and a sensitivity of 84.8% (28/33). The rapid tests were in substantial agreement between the two labs with a kappa of 0.74 and a concordance rate of 87.5%.

**Conclusion:**

Real-time performance of the NCF-based rapid test was comparable to controlled research conditions, supporting its feasibility for integration into routine microbiology laboratory workflows. Further validation is needed to confirm these results and to clarify the clinical significance of early detection of CzIE in MSSA.

**Disclosures:**

Jose M. Munita, MD, MSD LATAM: Grant/Research Support|Pfizer, Inc.: Grant/Research Support Cesar A. Arias, MD/PhD, UptoDate: Royalties

